# Effects of dexmedetomidine on delirium duration of non-intubated ICU patients (4D trial): study protocol for a randomized trial

**DOI:** 10.1186/s13063-018-2656-x

**Published:** 2018-06-04

**Authors:** Clémence Louis, Thomas Godet, Gérald Chanques, Nathalie Bourguignon, Dominique Morand, Bruno Pereira, Jean-Michel Constantin, for the AZUREA network

**Affiliations:** 1 Département de Médecine Périopératoire (MPO), Centre Hospitalier Universitaire (CHU) Clermont-Ferrand, 63003 Clermont-Ferrand, France; 2 GReD; UMR/CNRS6293; Université Clermont-Auvergne; INSERM U1103, 63003 Clermont-Ferrand, France; 3 Département d’Anesthésie-Réanimation B, Hôpital Saint-Eloi, Centre Hospitalier Universitaire (CHU) Montpellier, 34090 Montpellier, France; 4 Centre Hospitalier Universitaire (CHU) Clermont-Ferrand, Délégation à la Recherche Clinique et à l’Innovation (DRCI), 63000 Clermont-Ferrand, France; 5 Département de Médecine Périopératoire (MPO), Hôpital Estaing, Centre Hospitalier Universitaire (CHU) Clermont-Ferrand, 1 place Lucie Aubrac, 63003 Clermont-Ferrand, France

**Keywords:** Dexmedetomidine, Haloperidol, Delirium, ICU patients, Sedation, Mechanical ventilation, Intubation

## Abstract

**Background:**

Delirium during intensive care unit (ICU) stay is frequent and associated with significant morbidity, mortality and healthcare-related costs. International guidelines suggest its prevention. However, curative treatment remains unclearly established. Despite contradictory and ambiguous academic literature, international guidelines suggest the use of second-generation (atypical) antipsychotics over haloperidol. However, haloperidol remains the most widely used neuroleptic worldwide as a first-line treatment of agitation and/or delirium. Dexmedetomidine, an alpha2-adrenergic receptors agonist, has shown its efficiency in the treatment of delirium in intubated patients but also in its prevention. Dexmedetomidine represents a widely used alternative to haloperidol. Only few studies have compared the efficacy of dexmedetomidine in non-intubated ICU patients as a first-line curative treatment of delirium. The main objective of the 4D trial is to demonstrate that dexmedetomidine decreases delirium duration compared to placebo.

**Methods/design:**

The 4D trial is an investigator-initiated, prospective, multicenter, randomized, double-blinded, two-arm trial, randomizing 300 non-intubated ICU patients with a diagnosis of agitated delirium to receive dexmedetomidine or placebo as a cure. In case of agitation (RASS≥ + 2), immediate haloperidol administration will be allowed, to protect patient and staff in charge, while waiting for study treatment action. The primary outcome measure is a composite of duration of agitation or delirium or the use of intubation with deep sedation and mechanical ventilation. Secondary outcomes include mortalities at 7 and 30 days, ICU length of stay and occurrence of adverse effects related to dexmedetomidine use (bradycardia or hypotension requesting any treatment; or haloperidol use (neuroleptic malignant syndrome, extrapyramidal syndrome, prolonged QTc). The sample size will allow the detection of a 50% decrease of agitation duration (120 min), of an absolute reduction of delirium duration (1 day) and of a 50% relative decrease of intubation and mechanical ventilation, with a type 1 error rate of 1.8% (error risk inflation due to components of composite) and power of 90%, assuming a 15% incidence of intubation and mechanical ventilation requirements, an agitation duration of 240 min and a delirium duration of 3 days. One hundred and ten patients by group will be needed. An intermediate analysis is scheduled and requires the inclusion of 150 patients.

**Discussion:**

The 4D trial may provide important data on the safety of commonly used sedative dexmedetomidine and could have a significant impact on future treatment of non-intubated ICU patients presenting with agitated delirium.

**Trial registration:**

ClinicalTrials.gov, ID: NCT 03317067. Registered on 23 October 2017.

**Electronic supplementary material:**

The online version of this article (10.1186/s13063-018-2656-x) contains supplementary material, which is available to authorized users.

## Background

Delirium, whatever its presentation (agitated or not), is a frequently observed condition in intensive care unit (ICU) patients. According to recent studies, incidence ranges between 16 and 89% [1, 2]. Diagnosis requires a clinical examination, which includes changes in consciousness (altered concentration, decreased attention), in cognitive function (disorganization of thought, temporal disorders, memory problems) or in perception (hallucinations). Delirium appearance is brutal and its natural evolution fluctuates over time. Different scores can help physicians to diagnose delirium, the most common being the Confusion Assessment Method for the ICU (CAM-ICU) [3–5] and the Richmond Agitation and Sedation Scale (RASS) [6, 7]. The CAM-ICU scale is a readily available and reliable instrument to monitor delirium in ICU patients. Delirium is defined in terms of four diagnostic features, and is deemed positive when Feature 1 (acute change or fluctuating course of mental status) and Feature 2 (inattention) and either Feature 3 (altered level of consciousness) or 4 (disorganized thinking) are present [5]. RASS allows the evaluation of agitation and sedation of ICU patients and is included in Feature 3 of the CAM-ICU. This validated 10-point Visual Numeric Scale Numeric Rating Scale ranges from − 5 to 4. The score definitions are as follows: − 5, unarousable; − 4, deep sedation; − 3, moderate sedation; − 2, light sedation; − 1, drowsy; 0, alert and calm; 1, restless; 2, agitated; 3, very agitated; 4, combative.

Although incidence overestimation is likely, delirium is difficult to diagnose, especially in its hypoactive (calm) presentation. Delirium remains a public health issue because its presence is associated with long-term adverse outcomes: decreased survival [8], increased ICU and hospital lengths of stays which are associated with increased healthcare-associated costs [9]. Adverse outcomes are even more important when delirium is severe and prolonged.

Several risk factors have been identified to date [10]. Patients’ characteristics (age), habits (current smoking status, drug addiction or alcohol abuse [11]), postoperative periods, prolonged hospitalization, treatment including benzodiazepines, patient isolation and ICU hospitalization have been associated with delirium occurrence. The North American guidelines proposed strategies to prevent delirium [12]. Once detected, the curative treatment of delirium remains uncertain to date. A non-pharmacological approach seems mandatory, including early rehabilitation, limited number of caregivers in charge of the patient or sleep-disorder prevention [12]. These non-pharmacological approaches are usually insufficient and a pharmacological approach is often necessary, especially for agitated (hyperactive) delirium.

Haloperidol, a centrally acting dopamine antagonist, is the most frequently used drug for delirium treatment because of its antipsychotic and sedative properties without anticholinergic effects [13]. Despite ease of use based on titration opportunity, a short delay of action, and intravenous administration, haloperidol carries potential adverse effects such as neuroleptic malignant syndrome, dyskinesia, extrapyramidal syndrome, excessive somnolence and deep sedation with potential apnea. However, the efficacy of this drug has not yet been demonstrated in the treatment [14] or the prevention [15] of delirium in ICU compared to placebo. In addition, the rate of non-responders to haloperidol may sometimes be high and requires the use of other drugs to jugulate agitation. In this latter case, other sedative neuroleptics or benzodiazepines can be used, even if these latter drugs might be directly responsible for maintaining delirium. In some cases, deep sedation and mechanical ventilation may be required to cure agitation and delirium.

Dexmedetomidine, an alpha2-adrenergic receptors agonist, could be an alternative to haloperidol [16, 17]. This drug has proven efficacy in the treatment of delirium in intubated ICU patients as well as delirium prevention [18, 19]. Dexmedetomidine combines several advantages: a moderate sedative action, a wide therapeutic index, the absence of infusion rates adaptation outside hepatocellular insufficiency and a substantial analgesic effect. In 2009, Reade et al. showed that using dexmedetomidine to treat delirium in ICU reduced delays to extubation and decreased the use of additional sedatives as well as ICU length of stay compared to haloperidol [18]. In the DahLIA study, the same group demonstrated that the addition of dexmedetomidine to standard care in ventilated ICU patients increased ventilator-free hours at day 7 in patients with agitated delirium compared to placebo [19].

However, few studies have compared the efficacy of dexmedetomidine to treat delirium in *non-intubated* patients. To our knowledge, only one non-randomized prospective controlled trial [20] has compared dexmedetomidine to haloperidol in non-intubated patients. In this Spanish study, patients were initially treated with haloperidol and secondarily with dexmedetomidine in case of haloperidol-resistant delirium. Patients who received both treatments had a shorter delirium time associated with fewer disorders of consciousness.

Benefits related to the use of dexmedetomidine to treat delirium in non-intubated ICU patients are potentially important. Therefore, the definitive aim of the 4D trial is to investigate reduction of delirium duration related to dexmedetomidine use in non-intubated ICU patients, compared to placebo, in a multicenter, randomized, controlled, double-blind study.

## Methods/design

### Ethics

Due to the specific medical condition of recruited patients (agitated delirium), an emergency inclusion procedure will be possible. Written pursuit consent will be obtained from all participants (once medical condition resolved) or their next-of-kin. The Institutional Review Board of the University Hospital of Clermont-Ferrand (France) approved the trial. By 7 June 2017, the study had been approved for all centers by a central ethics committee (Comité de Protection des Personnes Sud-Est V, Grenoble, France, 17-CHCF-02) with the registration number EudraCT 2017–000731-14. Any further additional important protocol modification will require the approval of a central ethics committee (Comité de Protection des Personnes Sud-Est V, Grenoble, France, 17-CHCF-02). The 4D trial was registered on 23 October 2017 at http://www.clinicaltrials.gov with trial identification number NCT 03317067.

### Trial design

The 4D trial is an investigator-initiated, national, multicenter, double-blind, parallel, randomized, controlled, two-armed trial with concealed allocation of non-intubated ICU patients with delirium, 1:1 to treatment of delirium using dexmedetomidine (Orion Corporation, Espoo, Finland) or placebo (0.9% sodium chloride).

### CONSORT Diagram and SPIRIT Checklist

**Fig. 1:**
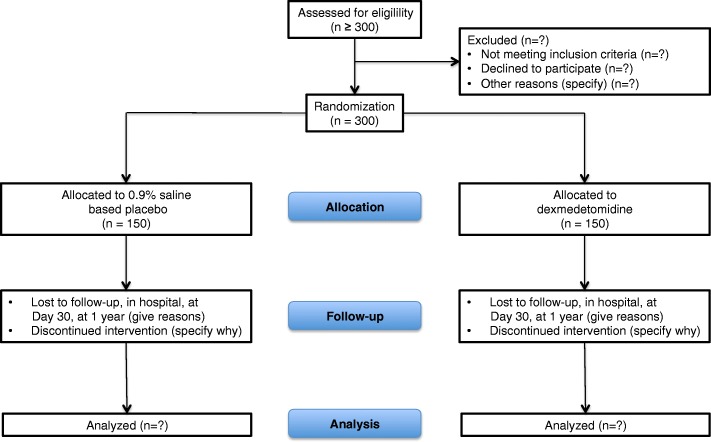
Consolidated Standards of Reporting Trials (CONSORT) flowchart illustrating the randomization and flow of patients in the study

**Fig. 2:**
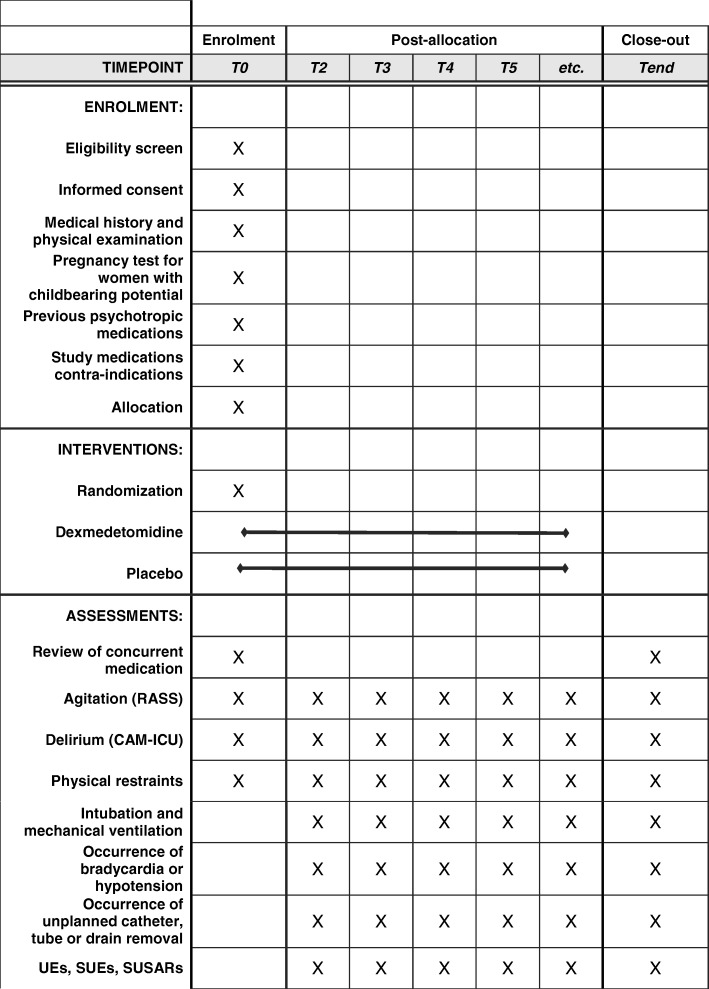
Patients’ schedule of activities according to the Standard Protocol Items: Recommendations for Interventional Trials (SPIRIT) Figure. *CAM-ICU* Confusion Assessment Method-Intensive Care Unit, *RASS* Richmond Assessment Sedation Scale, *SUE* serious unexpected events, *SUSAR* severe unexpected serious adverse event, *UE* unexpected events

The Consolidated Standards of Reporting Trials (CONSORT) Diagram of 4D is presented in Fig. 1. The Standard Protocol Items: Recommendations for Interventional Trials (SPIRIT) Figure and Checklist are available as Fig. 2 and Additional file 1, respectively.

### Selection of patients

Patients will be included in the 4D trial if they comply with the indicated inclusion and exclusion criteria as below.

#### Inclusion criteria

For inclusion, adult patients must meet all the following criteria:

1. Age > 18 years
2. Patient hospitalized in an ICU
3. Presenting a productive delirium according to the following criteria:
  - Acute onset (< 2 h) and fluctuating course during the same day
  - Alteration of cognitive functions: disorganization of thought (delirium of persecution, inability to reason logically), abnormal perceptions (hallucinations), memory impairments, temporal disorientation, non- or misrecognitions, difficulties in naming objects or writing)
  - In whom a simple cropping and non-medicated therapeutics are not sufficient to allow symptom resolution for a few hours
  - CAM-ICU (Annex 1, Additional file 1) positive *and* a RASS > + 1 (Annex 2, Additional file 1)
4. Non-intubated or extubated (> 24 h)
5. Tracheotomized without pressure support (> 24 h)
6. No contraindication to dexmedetomidine or haloperidol use

#### Non-inclusion criteria

Patients will not be included for any of the following reasons:

1. Administration of dexmedetomidine and/or clonidine and/or haloperidol during the 72 h before inclusion
2. Contraindication to the use of dexmedetomidine and haloperidol (history of allergy, Parkinson’s disease, oro-pharyngeal dysfunction, arterial hypotension or bradycardia, QTc interval prolongation, and hepatic or renal dysfunction), as mentioned in the Summaries of Product Characteristics
3. Neuropsychiatric pathology judged by the investigator as a potential source of bias (in particular: active drug addiction, psychosis, etc.)
4. Parturient or breast-feeding woman
5. Protected major (guardianship)
6. Patient’s or relative’s refusal to participate

### Randomization and blinding

Randomization will be conducted over a dedicated, password-protected, SSL-encrypted website (Clinsight Software) to allow immediate and concealed allocation. Each patient will be given a unique patient number and a randomization number. The allocation sequence will be generated by permutation blocks of 2. The participant allocation will be carried out by local investigators who will log into the randomization system using a personal ID code and will enter any relevant information (including weight to calculate infusion rates of study drugs).

Trial drugs (dexmedetomidine and 0.9% sodium saline) are not visually identical. However, to ensure the blinding of study drug administration, opaque bags will be available, each of those containing a medication vial. Trial bags will be blinded and identified only by a unique number. The initial allocation of trial drugs will be determined by the web-based randomization system through the allocation of a single bag number. Preparation of the study drug syringe will be conducted by a nurse and/or a physician independent of the study protocol and not in charge of the included patient. Further needs for study drugs (new syringe preparation) will be obtained from the web-based randomization system and the new allocation of a bag number. The logistics of the trial bags’ distribution to each of the 13 participating centers that are anticipated to be recruiting will be coordinated by the pharmacy of the coordinating center. The receipt, storage and dispensing of the blinded trial bags will be conducted by the pharmacy department in each individual trial site. Each trial site will have a sufficient number of sets of trial drugs to be allocated to the included patients. The initial and any subsequent allocation of trial drugs will be determined by the web-based randomization system at each site. This will ensure that the patient only receives the trial drug that they were randomized to receive. The information regarding which codes correspond to what treatment will be maintained in a secure location at the coordinating center. All staff, excluding the only person who prepared drug administration, at the participating trial sites and the coordinating center will be blinded to the treatment allocation. By the end of study protocol, unused medication bags as well as used medications bags and vials will be returned to the pharmacy of coordinating center.

No data monitoring committee (DMC) is programmed. In the present clinical study, patients are treated for a relatively short time and the drugs under investigation are well characterized and known for not harming patients [21].

### Trial interventions

After inclusion and randomization, patients presenting with a RASS ≥ + 2, will be intravenously administered boluses of haloperidol (2.5 mg), to promptly protect the patient against self-inflicted physical damages. Indeed, dexmedetomidine requires infusion of at least 1 h to obtain complete efficacy, incompatible with possibly aggressive and unsafe delirious patients.

**Fig. 3:**
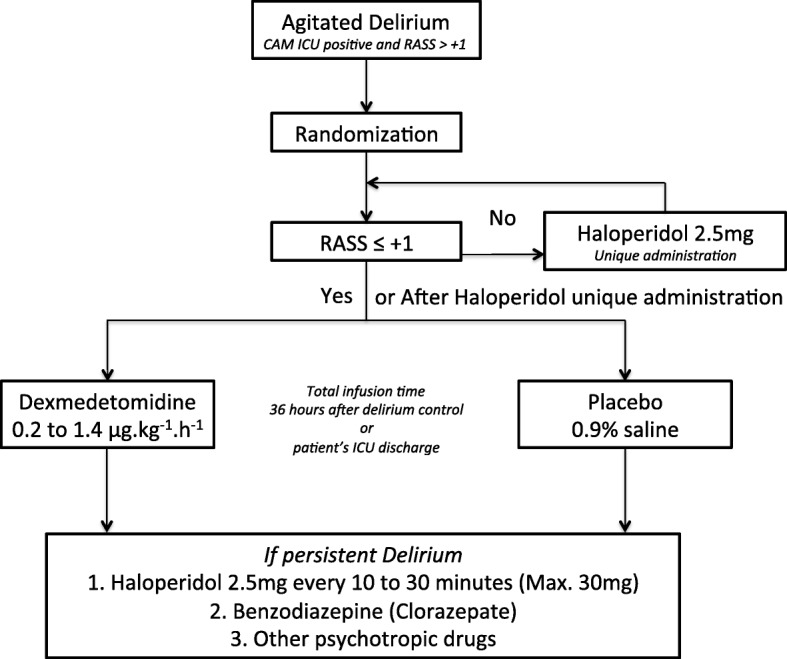
Study protocol diagram. Trial medication administration according to patient’s RASS. *ICU* intensive care unit, *RASS* Richmond Agitation Sedation Scale

After this bolus administration, or if RASS ≤ + 1, all included patients will be allocated to one of the following two study groups (Fig. 3):

1. Dexmedetomidine: patients in this group will receive dexmedetomidine infusion starting with a dose of 0.2 to 0.5 μg.kg^− 1^.h^− 1^
2. Placebo: patients randomized to placebo group will be administered an identical infusion of 0.9% sodium saline at an equivalent rate

Study medication will be adjusted by the bedside nurse or clinicians between 0.2 and 1.4 μg.kg^− 1^.h^− 1^ depending on the control of agitation. Continuous infusion will be continued at least 36 h after delirium disappears, or until the patient leaves the ICU if necessary.

If study medications are not sufficient to control delirium beyond the maximal dose, new boluses of haloperidol will be repeatable every 10 to 30 min up to the resolution of agitation (with a maximum dose of 30 mg). If maximal doses are reached, the use of benzodiazepines will be left to the discretion of physicians in charge of the patient (promoting clorazepate). Otherwise, the use of other psychotropic drugs will be documented.

### Summary of outcomes

The primary outcome measure will be a composite of:

1. Duration of agitation (in hours), defined by a RASS ≥ + 1
2. Duration of delirium (in days), defined by a positive CAM-ICU
3. Requirement of intubation to control delirium with deep sedation and mechanical ventilation

In addition, each component of the primary outcome measure will be analysed separately.

RASS will be evaluated at H1, H2, H3, H4, H5, and H6, after randomization and then every 12 h after inclusion;

CAM-ICU will be evaluated at H0 and then every 12 h;

If mechanical ventilation is necessary, the time between inclusion and intubation will be noted.

Secondary outcomes will be as follows:

- Length of ICU stay (in days)
- Number of ventilator-free days at day 30
- Adverse effects such as the occurrence of pneumonia (following the ATS definitions) and/or septicemia
- Duration of mechanical restraint prescribed and carried out
- Occurrence of bradycardia or rhythm disorders or myocardial ischemia or tachycardia or prolonged corrected QTc interval on the electrocardiogram, respiratory distress or apnoea
- Occurrence of hypotension requiring any vasopressor administration or hypertension
- Occurrence of dyskinesia, extrapyramidal symptoms or neuroleptic malignant syndrome
- All-cause mortality at day 7 and day 30
- Self-evaluation of sleep quality on a digital scale (0 to 10)
- SF36 score at 1 year
- Occurrence of delirium-related complications such as unplanned catheter, tube or drain removal

### Patient withdrawal

Studied drugs are to be used during ICU stay and for 36 h after delirium control. Nevertheless, a participant or a patient’s relative who no longer agrees to participate in the clinical trial can withdraw the informed consent at any time without need of further explanation. Patients who are withdrawn from the protocol will be followed up and analysed as with the remaining patients. In order to conduct intention-to-treat analyses with as little missing data as possible, it is in the interest of the trial to collect as much data from each participant as possible. Therefore, the investigator may ask the participant and/or relatives which aspects of the trial they wish to withdraw from (participation in the remaining follow-up assessments or use of already collected data) and, whenever possible, the participant will be asked for permission to obtain data for the primary outcome measure. If this person declines, no more data will be collected, and new patients will be randomized to obtain the full sample size. All randomized patients will be reported, and all data available with consent will be used in the analyses. If appropriate, missing data will be handled in accordance with multiple imputation procedures if missing data are greater than 5% [22–24].

### Safety

All adverse events thought to be related to the trial medications will be reported to the trial coordinating center. According to the French Public Health Code, all suspected unexpected serious adverse events will be reported to the ANSM. The investigator can use, after consultation of the Steering Committee, at any moment and in any situation that seems necessary (occurrence of severe adverse events), the unblinding procedure by consulting the web-based randomization table.

### Statistics

#### Sample size estimation

The sample size estimation is based on work proposed by O’Brien concerning composite endpoints [25]: weighted summation of single endpoints with standard procedures leads to asymptotically normal statistics. The average z-score extends this approach to include continuous, ordinal, dichotomous, and time-to-event endpoints. Specifically, continuous, ordinal, and dichotomous variables are converted to z-scores by subtracting an individual’s value from the overall mean and dividing by the SD of the pooled group; time-to-event variables are first transformed to log-rank scores and then converted to z-scores by subtracting the mean and dividing by the SD of the pooled data. The z-scores are then aligned to the same direction so that worse outcomes have smaller scores. The z-scores are then averaged across endpoints for each patient. Treatment groups will be compared with respect to this average z-score, the primary efficacy outcome. According to the academic literature concerning non-intubated patients in ICU for the duration of agitation (240 min, relative expected difference of 50%), the duration of delirium (3 days, absolute difference of 1 day) and proportion of patients requiring deep sedation with mechanical ventilation (15%, relative expected difference of 50%), *n* = 110 patients per group are required for a type I error at 0.018 (correction due multiple components of composite outcome) and a statistical power at 90%. Finally, we have decided to include 150 patients per group, i.e., 300 patients.

As sample size estimation was based on a composite criteria for which each of the expected effect sizes associated to each component could be re-evaluated, and considering that the correlation between components is not known, it appears reasonable and relevant to consider an interim analysis after the inclusion of 150 patients (75 by group). A difference between randomization groups will be considered statistically significant for a type I error at 0.01 (Kim-DeMets correction).

### Statistical analysis

Statistical analyses will be conducted using Stata software (version 13, StataCorp, College Station, TX, US). A two-sided *p* value of less than 0.05 will be considered to indicate statistical significance (except interim analysis).

Concerning the primary outcome, the comparison between groups will be analysed using Student’s *t* test or the Mann-Whitney *U* test. Normality will be studied by the Shapiro-Wilk test and homoscedasticity using the Fisher-Snedecor test. Intention-to-treat analysis will be considered for the primary outcome. Then, the analysis of the primary outcome will be completed by multivariable analysis using a linear mixed model to take into account: (1) fixed effects covariates determined according to univariate results and to clinical relevance and (2) center as random effects (to measure between and within-center variability). Results will be expressed as regression coefficients and 95% confidence intervals. Other continuous parameters outcomes will be analysed as described previously.

Categorical parameters (as proportion of patients with bradycardia and rhythm disorders, proportion of patients with hypotension requiring treatment with vasopressors, mortality) will be analysed using chi^2^ or Fisher’s exact tests in univariable analysis. Multivariable analysis will be performed using generalized linear mixed-model analysis (logistic for dichotomous dependent outcomes). The censored data (survival at day 30) will be estimated by the Kaplan-Meier method and compared by the log-rank test in the univariate situation and the marginal Cox model in the multivariate situation. The covariates defined above for the main analysis will be considered in a similar way, in addition to the center effect as random effect. Longitudinal analyses concerning repeated measures will be studied using random-effect models (linear or generalized linear), to take into account patient as random effect (slope and intercept), nestled in center random effect. According to clinical relevance and to European Medicines (EMA) and Consolidated Standards of Reporting Trials (CONSORT) recommendations, subgroup analyses will be proposed after the study of randomization group interaction in regression models (for repeated data or not). Secondarily, a per-protocol analysis will be considered. A post hoc subgroup analysis of elderly patients (above 65 years of age) will be conducted to investigate age influence. This threshold of 65 years was based on a recently published review on delirium in older patients [26]. In the event that investigators or reviewers introduce analyses in addition to those described above, these will be clearly delimitated as post hoc and will be considered hypothesis generating.

Finally, a sensitivity analysis will be performed and the nature of missing data will be studied (missing at random or not). According to this, the most appropriate approach to the imputation of missing data will be proposed.

### Data registration

Data will be entered into the web-based electronic case report form (eCRF) using Clinsight electronic data capture tools hosted at Centre Hospitalier Universitaire (CHU) Clermont-Ferrand by trial or clinical personnel under the supervision of the trial site investigators at each participating center. Data collection will be monitored by trained research coordinators.

The following data will be registered:

1. Pre-randomization and baseline characteristics: demographic data (age, height, weight, gender, and Body Mass Index); co-morbidities (hypertension (Y/N), renal dysfunction (Y/N), chronic heart failure (Y/N), heart-rhythm disorder (Y/N), diabetes mellitus (Y/N), malnutrition (Y/N), chronic alcoholism (Y/N) and active smoking (Y/N)); and routine biological data, including baseline serum creatinine and liver enzymes and metabolic acidosis.
2. Simplified Acute Physiology Score (SAPS II) score [27] and Sequential Organ Failure Assessment (SOFA) score [28]
3. Date of ICU admission (day, hour)
4. Reasons for ICU admission: postoperative period or medical or trauma-related
5. Delirium characteristics: delirium of persecution (Y/N), inability to reason logically (Y/N), abnormal perceptions or hallucinations (Y/N), temporal disorientation (Y/N), non- or misrecognitions (Y/N), difficulties in naming objects (Y/N) or writing (Y/N), fluctuating course during the same day (Y/N)
6. Date/hour of symptom onset
7. Time between admission to ICU and enrollment (hours)
8. CAM-ICU score [3]
9. RASS score [6]
10. BPS NI score [29] or Visual Analogic Scale (VAS) score for pain

At randomization, the following data will be collected:

1. Date/hour of randomization
2. Time between admission to ICU and randomization (min)
3. CAM ICU and RASS scores at H0
4. Behavioural Pain Scale in Non-intubated patients (BPS NI) score or VAS score at H0
5. Patient’s treatment and use of pharmacotherapy: morphine or opioids (Y/N), vasopressor drugs (Y/N): type and infusion rates, antipsychotic (Y/N), benzodiazepines (Y/N), antidepressant drug (Y/N)

Daily from randomization (08.00) to day 7 (or hospital discharge):

1. Daily lowest values for heart rate, blood pressure, peripheral O_2_ saturation, respiratory rate, temperature
2. Daily blood glucose level, blood pH, results of samples of plasma creatinine and creatinine clearance, plasma lactate, C-reactive protein (CRP), bilirubin, liver enzymes
3. RASS score at H1, H2, H3, H4, H5 and H6 after randomization and then RASS score every 12 h after randomization
4. CAM ICU score every 12 h after randomization
5. BPS NI score or VAS every 12 h
6. Cumulative dose of haloperidol at H1, H2, H3, H4, H5, H6 and then every 12 h
7. Minimum and maximum dose of dexmedetomidine or placebo infusion in μg.kg^− 1^.h^− 1^ every day
8. Total volume of study drug administration (μg.kg^− 1^) per day
9. Time to achieve RASS = 0, date, interval between randomization and achievement in hours
10. Time to achieve a negative CAM ICU score, date, interval between randomization and achievement in hours
11. Occurrence of intubation to treat delirium (Y/N), and date of diagnosis
12. Occurrence of hypotension (Y/N), hypertension (Y/N), bradycardia (Y/N), tachycardia (Y/N), rhythm disorders (Y/N), myocardial ischemia (Y/N) after study drug onset
13. Occurrence of hypotension requiring any vasopressor administration, date, vasopressor drug used, maximal dose, total dose and duration) after study drug onset
14. Occurrence of respiratory distress (Y/N) or apnoea (Y/N) after study drug onset
15. Patients receiving rescue drug (clorazepate) because of maximum tolerated dose is reached (Y/N, type, dose, duration)
16. Daily self-evaluation of sleep quality on digital scale if possible (Y/N and 0 to 10)
17. Length of ICU stay
18. Date of hospital discharge
19. Death (Y/N and date)

Thirty days after randomization:

1. Length of ICU stay (in days)
2. Date of hospital discharge (as obtained from hospital notes)
3. Survival status (If the patient is deceased, date of death)

If the patient is still present on day 30, follow-up will continue until hospital discharge:

1. Occurrence of intubation to treat delirium (Y/N and date)
2. Duration of mechanical restraint prescribed and carried out if it occurs
3. Number of ventilator free days at day 30
4. Survival status: alive (Y/N), if the patient is deceased, date of death

At 1 year after randomization:

1. Survival status (if the patient is deceased, date of death)
2. Short Form Health Survey, 36 items (SF36) score

### Data handling and retention

Data will be handled according to French law. All original records (including consent forms, reports of suspected unexpected serious adverse events, and relevant correspondences) will be archived at the trial sites for 15 years. The clean trial database file will be anonymized and maintained for 15 years.

### Enrollment and timeline

The patients are expected to be included from 13 French university and non-university hospitals during a 1-year period starting in December 2017.

Listing of trial centers at study initiation is presented below:

- CHU de Clermont-Ferrand
- Hospices Civils de Lyon, service d’anesthésie-réanimation
- CHU de Montpellier, hôpital St-Éloi, service de réanimation chirurgicale DAR B
- AP-Hôpitaux de Marseille, service de réanimation
- CHU de Nîmes, service de réanimation chirurgicale
- CHU de Nantes, service d’anesthésie-réanimation chirurgicale
- Centre Hospitalier Le Puy-en-Velay, service de réanimation
- Centre Hospitalier de Montluçon, service de réanimation
- Centre Hospitalier d’Aurillac, service de réanimation
- CHU de Nice, service d’anesthésie-réanimation
- CHU de Grenoble, service d’anesthésie-réanimation
- CHU de Saint-Etienne, service d’anesthésie-réanimation
- CHU de Tours, service d’anesthésie-réanimation

Each participating center has to include five patients per month (holidays excluded) to complete inclusions in less than 1 year.

2016–2017: protocol, approvals from the Ethics Committee, and trial tool development (eCRF, randomization system).

2018 to 2019: inclusion of patients.

2020: cleaning and closure of the database

Mid-2020: data analyses and writing of the manuscript, and submission for publication

### Publication plan

The trial is registered at http://www.clinicaltrials.gov. Upon trial completion, the main manuscript will be submitted to one of the major clinical journals regardless of the results. All trial sites, including patients, will be acknowledged, and all investigators at these sites will appear with their names under “the 4D investigators” in an appendix to the final manuscript. The 4D trial Steering Committee will grant authorship in adherence to the Vancouver guidelines and number of patients enrolled by the individual investigator. If a trial site investigator is to gain authorship, the site has to include 10 patients or more. If the site includes 20 patients or more, two authorships will be granted. A writing committee will be composed of members of the Steering Committee and investigators to define the order of authors of any publications.

The listing of authors will be as follows: T Godet (Clermont-Ferrand site investigator) will be first author, C Louis (Clermont-Ferrand site investigator) will be second author, then other members of the Steering Committee and trial site investigators depending on the number of included patients per site and per month (trial site investigator with the greater number of inclusion per month will be third author), B Pereira (biostatistician) will be third to last author, G Chanques (associated investigator) will be second to last author, JM Constantin (principal investigator) will be responsible for the writing of the manuscript and will appear as the last author and then ‘for AZUREA Network’ will be added.

### Finances

The 4D trial is funded by an institutional grant from Clermont-Ferrand University Hospital and a grant from AZUREA network (www.azurea.org).

Management and logistic of the trial drug distribution to each of the 13 participating centers that are anticipated to be recruiting will be coordinated using the web-based randomization system by the pharmacy of the Clermont-Ferrand University Hospital. The receipt, storage and dispensing of the blinded trial drugs will be the responsibility of the pharmacy department in each individual trial site. This will be performed in accordance with accredited standards for routine pharmacy practice.

Funding sources have no influence on trial design, trial conduct, data handling, data analysis or writing of the manuscript.

### Perspectives

Millions of patients undergo ICU admission worldwide each year. Delirium occurrence is frequent and is associated with increased patients’ short- and long-term morbidity and mortality as well as healthcare-associated costs. Few studies have examined the effects of dexmedetomidine in the treatment of agitated delirium in non-intubated ICU patients and much of the available data are extrapolated from mechanically ventilated patients. As far as the investigators are aware, no other large RCTs are assessing the efficacy and safety of dexmedetomidine or placebo with the goal of delirium treatment in non-intubated ICU patients.

## Discussion

Performing the 4D trial is in line with conclusions from the 2013 recommendations of the American College of Critical Care Medicine on the clinical practice guidelines for the management of pain, agitation and delirium in adult patients in the ICU [12], especially in those not requiring tracheal intubation and mechanical ventilation.

Dexmedetomidine is widely used in ICU patients as a component of sedation therapy for delirium treatment or prophylaxis. However, haloperidol remains the most widely used treatment of agitation and delirium in ICU [30, 31], but may reveal ineffective and associated with potential cardiovascular complications. The low level of evidence of the few studies on the subject encouraged us to conduct the 4D trial, and to compare dexmedetomidine to placebo since haloperidol, and perhaps second-generation antipsychotics, should not remain first-line delirium treatments in non-intubated patients as stated in 2013 guidelines on agitation, pain and delirium [12]. Indeed, recent trials have reported inefficiency of haloperidol, when compared to placebo, to cure delirious ICU patients [14] and to prevent delirium appearance in high-risk patients [15]. Carrasco and colleagues [20] investigated dexmedetomidine for the treatment of hyperactive delirium refractory to haloperidol in non-intubated ICU patients. The trial concluded to a potential cost-benefit of dexmedetomidine over haloperidol in this non-RCT. However, this trial compared two groups: haloperidol alone versus dexmedetomidine *plus* haloperidol (since the mean elimination half-life is 21 h) with dexmedetomidine as a second-line treatment. Important information from the trial by Carrasco et al. is the excellent tolerance of co-administration of dexmedetomidine and haloperidol. As reported by these authors, dexmedetomidine could be the medication of choice to treat delirious patients due to specific properties: absence of excessive sedation, easy titration, fewer side effects than neuroleptics and rare interactions with other drugs.

Whatever the result of the 4D trial, this will provide needed and new data on the efficacy and safety of commonly used medications, which could have a significant impact on future treatment of non-intubated ICU patients presenting with delirium.

## Trial status

The 4D trial is currently recruiting patients.

## Additional file

Additional file 1: Standard Protocol Items: Recommendations for Interventional Trials (SPIRIT) 2013 Checklist: recommended items to address in a clinical trial protocol and related documents. (DOCX 571 kb)

## Abbreviations

BPS NI: Behavioural Pain Scale in Non-intubated patients
CAM ICU: Confusion Assessment Method for the ICU
CHU: Centre Hospitalier Universitaire (University Hospital)
eCRF: Electronic (web-based) case report form
ICU: Intensive care unit
RASS: Richmond Agitation Sedation Scale
SAPS: Simplified Acute Physiology Score
SOFA: Sequential Organ Failure Assessment
VNS: Visual Numeric Scale
